# Healthy lifestyle factors and male perpetration of intimate partner violence: a cross-sectional study in Mwanza, Tanzania

**DOI:** 10.1080/16549716.2024.2397842

**Published:** 2024-09-13

**Authors:** Anna E. Jacob, Gerry Mshana, Neema Mosha, Ramadhan Hashim, Simon Sichalwe, Donati Malibwa, Saidi Kapiga, Philip Ayieko, Heidi Stöckl

**Affiliations:** a Institute for Medical Information Processing, Biometry and Epidemiology, LMU Munich, Munich, Germany; b Pettenkofer School of Public Health, LMU Munich, Munich, Germany; c National Institute for Medical Research, Mwanza, Tanzania; d Mwanza Intervention Trials Unit, Mwanza, Tanzania; e Infectious Disease Epidemiology Department, London School of Hygiene & Tropical Medicine, London, UK

**Keywords:** Interpersonal violence, diet, sleep, exercise, lifestyle factors, young men, Africa

## Abstract

**Background:**

In Tanzania, nearly half of ever-married women have experienced some form of intimate partner violence (IPV), yet little knowledge of IPV from the male perspective exists.

**Objective:**

To explore the role of essential healthy lifestyle factors, diet, sleep, and exercise, and their potential role in IPV perpetration.

**Methods:**

A cross-sectional survey was conducted with 1,002 young men (ages 18–24), 754 of which were in an intimate relationship in the previous year. The study took place in Mwanza, Tanzania and used multivariable logistic regression models to explore associations between male perpetration of IPV and diet, sleep, and exercise.

**Results:**

Six types of IPV perpetration were investigated separately and the prevalence of controlling behaviours (79.4%), economic abuse (30.6%), emotional abuse (47.3%), physical violence (16.4%), sexual violence (23.3%), and combined physical and/or sexual violence (32.1%) were obtained. Regular exercise demonstrated a protective effect for economic abuse perpetration; the chance of mildly active individuals perpetrating economic abuse was 38% less than their inactive counterparts (*p* = 0.003). Associations with sleep were varied and did not show a clear directional relationship. Diet, defined as poor food variety, was positively associated with every IPV type except physical violence and was significant in sexual violence perpetration (aOR:1.57, 95%CI:1.21–2.05).

**Conclusions:**

The results from this study indicate that considering healthy lifestyle behaviours – diet, sleep, and exercise – in the design of intervention programmes may be beneficial in reducing IPV perpetration in Tanzania, and that they should be considered alongside previously established evidence-based risk factors.

## Paper Context

- **Main findings:** Physical healthy lifestyle factors may play a weak role in male-perpetrated intimate partner violence but may not be as important as mental health and relationship dynamics.
- **Added knowledge:** Limited information exists on potential causes and pathways of male-perpetrated intimate partner violence.
- **Global health impact for policy and action:** The results presented can aid in guiding the development of future intimate partner violence interventions and prevention strategies.

## Background

Intimate partner violence (IPV) is a serious global public health concern. While the focus has largely been on women’s experience of IPV, investigation of the male perpetrator perspective is only slowly growing [1,2]. IPV is defined as any action between intimate partners that results in physical, psychological, or sexual harm to those in the relationship [3]. According to a recent systematic review, the global prevalence of women aged 15–49 experiencing physical and/or sexual violence in their relationships was 27%, with the highest rates of IPV occurring in Oceania (excluding Australia and New Zealand) and sub-Saharan Africa [4]. While physical and sexual IPV are well known, emotional IPV, economic IPV, and controlling behaviours also warrant attention [3,5]. Detrimental effects of IPV on those affected and their families can be both short- and long-lasting, resulting in social and economic costs to individuals, governments, and other involved organisations [4,6]. As the COVID-19 pandemic appears to be associated with increased IPV rates, reducing the economic cost, social burden, and negative physical and mental health consequences of IPV are pressing priorities for both pandemic recovery and achieving the United Nations’ Sustainable Development Goal 5 on gender equality and empowerment for all [7,8]. Reaching these targets will require effective, evidence-based intervention measures, which must involve male partners and be tailored to the specific community context [9]. Identifying and investigating potential risk factors for male intimate partner violence perpetration, such as physical health, is paramount in developing effective intervention strategies.

In eastern sub-Saharan Africa, the prevalence of women experiencing physical and/or sexual IPV is about 38% [1,4]. In Tanzania, specifically, the past-year prevalence in 2018 was 24% and may be as high as 46% when considering emotional violence in addition to physical and sexual IPV during the lifetime [4,10]. The prevalence in Mwanza appears even higher with estimates of 61% and 27% for lifetime and the past 12 months, respectively [11]. Forms of IPV beyond sexual and physical violence have seen little investigation in the context of sub-Saharan Africa, and controlling behaviours, economic abuse, and emotional abuse may play major roles in IPV consequences while not always co-occurring with physical or sexual violence [9,11,12].

Young men were the focus of this study for a variety of reasons, including the overall young demographic of Tanzania and the commonality and expectation of young marriage. Additionally, young adults are an at-risk group for IPV perpetration and could be an ideal demographic to target interventions. They are also often an overlooked group in IPV investigations, which tend to favour adult population samples [13,14].

Data available on male perpetration experiences are more limited than female experiences; however, some figures have been previously published. In Sri Lanka, 24% of men reported perpetrating physical violence, 20% reported sexual violence, and 36% reported physical and/or sexual violence against a partner [3,15]. In the same study, 41% of men reported perpetrating emotional abuse and 18% reported economic abuse against a partner [3,15]. Some known risk factors of IPV perpetration have demonstrated differences between males and females with alcohol and substance use, childhood abuse, and witnessing parental IPV all being stronger indicators for men [16]. Additional known male risk factors include poverty, education level, age, relationship practices, mental health, insomnia, and history of violence in the family and childhood [3,9,17]. Migration status may also be a risk factor for violence due to possible lack of social cohesion in these populations and increased rates of overall violence [18]. Because data reflecting the male perspective of male-perpetrated IPV are limited, especially in low-middle income countries (LMICs), identifying male-specific risk factors is essential to provide evidence for effective intervention strategies.

The need for upstream, primary prevention of IPV is clear and various interventions have already been implemented to address some of the root causes of IPV, including societal acceptance of violence and gender inequality [2]. Tailoring interventions to the specific community and setting in which they are being used is also necessary due to the complex cultural and societal nuances associated with IPV [9]. Previous research and individual perpetration interventions have included the mental health aspect of IPV, which remains important and should always be considered in combination with any physical risk factors identified [9,16]. Since mental and physical health are implicitly connected, the goal of this analysis was to shift the focus to more tangible factors that are associated with good physical health – diet, sleep, and exercise – and their possible connections to IPV perpetration. This relationship has not yet been investigated but may be an easier target in future interventions as physical health goals and programmes may be more concrete and implementable compared to mental health targets alone.

**Figure 1.**
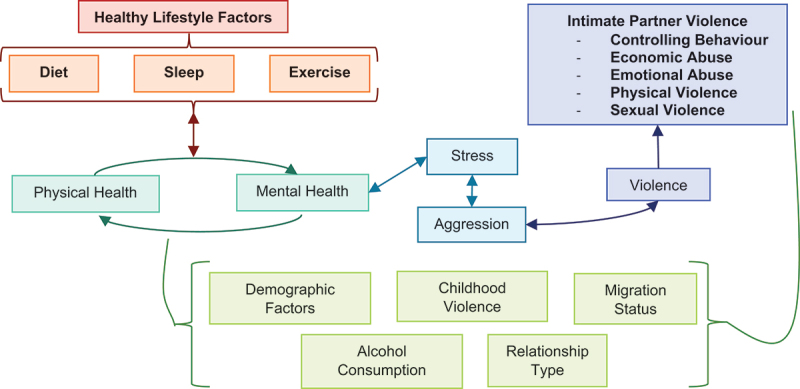
Conceptual framework connecting healthy lifestyle factors of interest to IPV perpetration.

Diet, sleep, and exercise are classic factors in maintaining good health [19]. Stress, anger, and aggression play into violent and outburst behaviours and are known markers of IPV perpetration [14,16,20,21]. The pathway from stress to violence and aggression is supported by the popular frustration-aggression theory, which posits that acts of aggression are preceded by frustration, and by recent biological evidence [22–24]. A healthy lifestyle is known to reduce stress levels and therefore may play an indirect role in the pathways of IPV perpetration, conceptualised visually in Figure 1 [25–27].

Indirect connections are illustrated through physical health/mental health and stress/aggression pathways. Items in light green are previously identified risk factors that may confound this relationship.

Food insecurity has received some attention in the literature and has been identified in association with female experience of IPV and as a potential contributor to male-perpetrated IPV [13,28,29]. It has also been linked to poor diet quality and related negative health outcomes, making poor food variety a suitable proxy for diet quality [29–31]. Sleep has received little investigation in this context, but is strongly connected with mental health and has demonstrated bidirectional pathways in IPV, both as a predictor and consequence, and as a mediator between IPV and mental/physical health [14,32,33]. Exercise, in connection with IPV, has not been previously investigated; however, there is consensus that an active lifestyle reduces stress and ensures better long-term outcomes than a sedentary lifestyle [34]. In a study of young boys in nine LMICs, a physically active lifestyle was associated with lowered risk of violence and injury, although these findings were not IPV-specific [35]. Diet, sleep, and exercise are the foundation of a physically healthy lifestyle and through indirect pathways may play a role in male perpetrated IPV in the sub-Saharan context. This study therefore sought to investigate the role of healthy lifestyle factors in men’s IPV perpetration.

## Methods

### Study design and procedures

A cross-sectional survey was conducted among 1,002 young men between June 2021 and April 2022 in Mwanza, Tanzania. Two urban districts of the Mwanza Region, Nyamagana and Ilemela, were included in the study. These districts are further divided into municipal wards, which were stratified into densely and sparsely populated areas and from each district, three wards of each type (densely and sparsely populated) were then randomly selected. A total of 24 streets were randomly selected from the sample of districts following GPS mapping of the streets. One hundred and twenty points were randomly generated, and the survey team visited the two houses closest to the points to inquire if any young man aged 18 to 24 lived in the house. If more than one eligible young man lived there, the participant was chosen randomly. After the survey was introduced and the participant provided consent, the questionnaire was administered.

Interviews were conducted face-to-face with trained male interviewers; an electronic tablet was used to record answers for sensitive questions regarding IPV-perpetration outcomes through Audio Computer-Assisted Self-Interviewing (ACASI) data collection. Interviews were conducted in private spaces by specially trained interviewers that followed strict protocols to ensure confidentiality and any needed support for participants.

The sample size calculation for the study was based on the investigation of changes in mental health, alcohol abuse, and a difference of 15% to 30% in sustainable income. With a conservative IPV prevalence estimate of 28%, a sample size of 1,000 men was determined to yield a power of over 90%. Men between the ages of 18 and 24 who resided in Mwanza, Tanzania and agreed to participate were included. The current investigation excluded any participants that had not been in an intimate relationship with a woman at some point in the 12 months preceding their interview. Questions were not tailored based on the type of relationship the participant reported.

### IPV outcomes

Controlling behaviour, economic abuse, emotional abuse, physical violence, sexual violence, and physical and/or sexual violence perpetration against an intimate partner were defined as committing one or more acts within the previous 12 months (Table 1). Questions in this section were derived from the Sonke CHANGE Trial Questionnaire, CoVAC Adolescent Questionnaire, and IMAGES Questionnaire 2011, while some were developed based on qualitative interviews [18,36,37]. All six binary variables formed the separate outcomes of interest in this study.

**Table 1.** Questionnaire items describing IPV outcomes.

Form of Intimate Partner Violence	Associated Questions
*Controlling Behaviour*	Thinking about your current or most recent or past wife/partner, would you say that you have: Insisted on knowing where she is at all times? Tried to restrict or reduce her having contact with her family or friends? Checked and controlled her mobile phone? Told her what to wear and how to behave?
*Economic Abuse*	Made important financial decisions without her? You ever prohibited her from getting a job, going to work, trading or earning money? You ever taken her earnings against her will?
*Emotional Abuse*	Spread rumours about her or tried to turn her friends against her? Spoken to her in a mean (hostile) tone of voice? Insulted her or deliberately said things to make her feel bad about herself? Made fun of her, belittled her or humiliated her in front of other people? Tried to scare or intimidate her on purpose, for example by the way you looked at her, by shouting, or by smashing things? Threatened to hurt or actually hurt people she cares about as a way of hurting her, or damage things of importance to her?
*Physical Violence*	Thinking about your current or most recent or past wife/partner, would you say that you have: ever slapped her, ever pushed or shoved her or thrown something at her that could hurt her? Ever hit her with a fist or something else that could hurt her, ever kicked, dragged, beaten, choked or burned her? Ever threatened to use or actually used a gun, knife or other weapon against her?
*Sexual Violence*	Ever forced her to have sex with you when she did not want to? Ever had sex with her when you knew she did not want it but you believed she should agree because she was your wife/partner? Ever forced her to do something sexual that she did not want to?

### Healthy lifestyle factors

The main predictors of interest were diet, sleep, and exercise. Diet in our context referred to poor food variety since diet was not measured independently. This was assessed based on a food insecurity question, ‘In the past [4 weeks], did it happen that you or any household member had to eat a limited variety of foods because of lack of resources?’, derived from the Household Food Insecurity Access Scale [38]. A positive response was indicative of poor food variety while a negative response was deemed sufficient variety. The question used to measure sleep was not from a validated questionnaire and simply asked participants how many hours per night they sleep on average. Responses were separated into four categories: 1–4 h, 5–6 h, 7–8 h, and 9+ h, with 7–8 h being the reference and deemed sufficient sleep [19]. Participants were also asked how many days per week, on average, they participated in vigorous physical activity for at least 10 min. This question was derived from the Mwanza Students Cohort 2017 Questionnaire and was included as a categorical variable based on apparent groupings of respondents: inactive (0–1 days/week), mildly active (2–3 days/week), moderately active (4–5 days/week), and very active (6–7 days/week).

### Covariates

The analysis controlled for various covariates identified as potential confounders in the pathways (Figure 1) [3,16,17,21]: age, measured in number of years, education level capturing primary incomplete and complete, secondary school complete, and post-secondary, employment in the last 12 months, whether they were migrants to the area (has not always lived in the area), consumption of alcohol in the last 12 months, relationship type (monogamous, casual, or not currently in a relationship), and childhood exposure to violence based on the Adverse Childhood Experience Score [39]. Mental health, aggression, and stress may all play a role in effect modification based on the conceptualised pathway (Figure 1) and were therefore not included as covariates. Income and household size were not included. Income in this context was difficult to objectively quantify due to cash in kind transfers and the possible impact of COVID-19.

### Statistical analysis

All analyses were conducted using RStudio software for statistics version 4.1.2 [40]. Outcomes, predictors, and covariates were first explored through univariate analysis presenting frequencies and proportions for categorical variables, and mean and standard deviation for continuous variables. Individual multivariable logistic regression models were built for each IPV outcome using only diet, sleep, and exercise variables as predictors (Model 1). Models were then built to additionally include age, education level, migration status, employment status, alcohol consumption, relationship type, and exposure to violence as a child (Model 2). Cluster-robust standard errors were calculated based on the street level to account for the cluster-based sampling method. Covariates were selected based on literature and previous knowledge, as recommended by Heinze et al. [41,42]. Odds ratios for models 1 (ORs) and 2 (aORs) were recorded for each outcome along with associated 95% confidence intervals (CI)s and p-values. For models where single categories were significant, a likelihood ratio test was performed to evaluate significance of the categorical variable in the overall model. The cut-off for statistical significance was α = 0.05. Sensitivity analyses were performed by altering the coding of exercise to continuous and sleep to dichotomous variables, independently, and noting the impact on model results (Supplementary Materials).

## Results

Interviews were conducted with 1,002 young men between the ages of 18 and 24 in Mwanza, Tanzania. Twenty-one men (2.1%) who were asked to interview chose not to participate and 227 (22.7%) were excluded as they were not in a relationship in the previous 12 months leaving 754 men (75.2%) included in the analysis. Demographic characteristics and healthy lifestyle variables of the sample were stratified by IPV perpetration type (Table 2). High levels of controlling behaviours (79.4%) and childhood experience of violence (>98.7%) were reported in the sample. There were no missing data for any participants for the variables included in this study.

**Table 2.** Study population characteristics stratified by IPV type.

	Total	Controlling Behaviour	Economic Abuse	Emotional Abuse	Physical Violence	Sexual Violence	Physical or Sexual Violence
*N (%)*	754	599 (79.4)	231 (30.6)	357 (47.3)	124 (16.4)	176 (23.3)	242 (32.1)
*Poor Food Variety (%)*	372 (49.3)	304 (50.8)	123 (53.2)	188 (52.7)	60 (48.4)	103 (58.5)	130 (53.7)
*Hours of Sleep/Night (%)*							
1–4	18 (2.4)	13 (2.2)	5 (2.2)	3 (0.8)	2 (1.6)	2 (1.1)	3 (1.2)
5–6	159 (21.1)	130 (21.7)	56 (24.2)	78 (21.8)	25 (20.2)	38 (21.6)	52 (21.5)
7–8	397 (52.7)	313 (52.3)	120 (51.9)	186 (52.1)	66 (53.2)	97 (55.1)	131 (54.1)
9+	180 (23.9)	143 (23.9)	50 (21.6)	90 (25.2)	31 (25.0)	39 (22.2)	56 (23.1)
*Exercise Habits (Days/Week) (%)*							
Inactive (0–1)	318 (42.2)	241 (40.2)	116 (50.2)	155 (43.4)	52 (41.9)	74 (42.0)	102 (42.1)
Mildly Active (2–3)	240 (31.8)	200 (33.4)	64 (27.7)	121 (33.9)	43 (34.7)	55 (31.2)	77 (31.8)
Moderately Active (4–5)	71 (9.4)	58 (9.7)	21 (9.1)	25 (7.0)	8 (6.5)	18 (10.2)	22 (9.1)
Very Active (6–7)	125 (16.6)	100 (16.7)	30 (13.0)	56 (15.7)	21 (16.9)	29 (16.5)	41 (16.9)
*Age* *(mean (SD))*	21.1 (1.9)	21.2 (2.0)	21.1 (2.0)	21.2 (2.0)	20.9 (2.0)	21.3 (1.9)	21.1 (2.0)
*Migrant to Mwanza (%)*	290 (38.5)	234 (39.1)	84 (36.4)	132 (37.0)	41 (33.1)	53 (30.1)	80 (33.1)
*Highest Education Level (%)*							
Primary Incomplete	87 (11.5)	58 (9.7)	25 (10.8)	36 (10.1)	23 (18.5)	19 (10.8)	33 (13.6)
Primary	287 (38.1)	220 (36.7)	90 (39.0)	133 (37.3)	52 (41.9)	72 (40.9)	97 (40.1)
Secondary	296 (39.3)	248 (41.4)	92 (39.8)	151 (42.3)	40 (32.3)	62 (35.2)	85 (35.1)
Post-Secondary	84 (11.1)	73 (12.2)	24 (10.4)	37 (10.4)	9 (7.3)	23 (13.1)	27 (11.2)
*Employed (%)*	573 (76.0)	455 (76.0)	187 (81.0)	273 (76.5)	100 (80.6)	142 (80.7)	192 (79.3)
*Relationship Status (%)*							
Cohabiting	90 (11.9)	73 (12.2)	28 (12.1)	49 (13.7)	22 (17.7)	20 (11.4)	33 (13.6)
Monogamous but not cohabiting	320 (42.4)	270 (45.1)	87 (37.7)	139 (38.9)	36 (29.0)	57 (32.4)	79 (32.6)
One or more casual partners	233 (30.9)	172 (28.7)	81 (35.1)	112 (31.4)	44 (35.5)	73 (41.5)	94 (38.8)
No current partner but had one in last twelve months	111 (14.7)	84 (14.0)	35 (15.2)	57 (16.0)	22 (17.7)	26 (14.8)	36 (14.9)
*Consumes Alcohol (%)*	189 (25.1)	159 (26.5)	76 (32.9)	108 (30.3)	48 (38.7)	59 (33.5)	82 (33.9)
*Experienced Violence During Childhood (%)*	744 (98.7)	594 (99.2)	230 (99.6)	355 (99.4)	123 (99.2)	176 (100.0)	241 (99.6)

### Intimate partner violence

Of the 754 participants, 599 (79.4%), 231 (30.6%), and 357 (47.3%) reported perpetrating controlling behaviours, economic abuse, and emotional abuse against their partners, respectively. Perpetration of physical and sexual IPV was lower with 124 (16.4%) admitting to physical violence and 176 (23.3%) admitting to sexual violence. The combined physical and/or sexual IPV category contained 242 participants (32.1%).

### Healthy lifestyle factors

Nearly half the sample identified poor variety in their food availability (49.3%), which was even higher among those reporting sexual IPV (59.0%). A similar pattern was observed with sufficient sleep where just over half of participants (52.7%) claimed they slept a sufficient 7–8 hours per night on average. Sleep patterns did not vary much across IPV types. Exercise patterns were a bit more varied, but a large proportion of the sample was inactive in everyday life (42.2%). Nearly a third of participants were mildly active (31.8%) while smaller proportions were moderately and very active. Inactive men were more likely to report economic IPV perpetration (50.2%), while very active men were less likely to report economic IPV perpetration (13.0%).

### Risk factors

Multivariable logistic regression models produced varying results (Table 3). None of the factors of interest were significantly associated with physical IPV or physical and/or sexual IPV. A protective effect was observed between mild exercise and economic IPV (aOR = 0.62; 95%CI 0.45–0.85), and inadequate (1–4 h) sleep and emotional IPV (aOR = 0.20; 95%CI 0.05–0.80). Performing a likelihood ratio test for the overall model contribution of exercise in economic IPV (Chi-square: 8.79, p-value: 0.032), and sleep in emotional IPV (Chi-square: 9.13, p-value: 0.028) indicated that both played a significant role. Young men with poor food variety had 57% higher odds of perpetrating sexual IPV (aOR = 1.57; 95% CI 1.21–2.05) compared to men with sufficient variety.

**Table 3.** Odds ratios for healthy lifestyle factors for each type of male perpetrated IPV.

Variables	Model 1 OR [95% CI]	*p*-value^†^	Model 2 OR [95% CI]	*p* value^†^
***Controlling Behaviour***				
*Hours of Sleep/Night*				
1–4	0.64 [0.25–1.63]	0.350	0.61 [0.24–1.55]	0.300
5–6	1.14 [0.67–1.92]	0.631	1.00 [0.61–1.66]	0.987
7–8 (ref)	1.00		1.00	
9+	1.04 [0.76–1.43]	0.794	1.06 [0.77–1.48]	0.705
*Diet Variety*				
Sufficient (ref)	1.00		1.00	
Poor	1.29 [0.87–1.90]	0.206	1.24 [0.82–1.85]	0.304
*Exercise Habits (Days/Week)*				
Inactive (0–1) (ref)	1.00		1.00	
Mildly Active (2–3)	1.58 [0.96–2.58]	0.070	1.58 [0.93–2.67]	0.090
Moderately Active (4–5)	1.41 [0.71–2.79]	0.325	1.36 [0.63–2.96]	0.431
Very Active (6–7)	1.29 [0.60–2.75]	0.513	1.18 [0.57–2.44]	0.660
***Economic Abuse***				
*Hours of Sleep/Night*				
1–4	0.91 [0.42–1.96]	0.801	0.91 [0.42–1.97]	0.814
5–6	1.29 [0.82–2.05]	0.274	1.29 [0.85–1.98]	0.235
7–8 (ref)	1.00		1.00	
9+	0.86 [0.55–1.35]	0.515	0.86 [0.54–1.36]	0.511
*Diet Variety*				
Sufficient (ref)	1.00		1.00	
Poor	1.25 [0.90–1.73]	0.183	1.20 [0.87–1.67]	0.262
*Exercise Habits (Days/Week)*^*◊*^				
Inactive (0–1) (ref)	1.00		1.00	
Mildly Active (2–3)	**0.61 [0.45–0.82]**	0.001*	**0.62 [0.45–0.85]**	0.003*
Moderately Active (4–5)	0.71 [0.34–1.49]	0.360	0.71 [0.33–1.52]	0.375
Very Active (6–7)	0.54 [0.28–1.03]	0.062	0.56 [0.30–1.07]	0.080
***Emotional Abuse***				
*Hours of Sleep/Night*^*◊*^				
1–4	**0.21 [0.05–0.82]**	0.024*	**0.20 [0.05–0.80]**	0.023*
5–6	1.07 [0.75–1.54]	0.708	1.04 [0.74–1.47]	0.814
7–8 (ref)	1.00		1.00	
9+	1.12 [0.76–1.66]	0.560	1.16 [0.80–1.68]	0.435
*Diet Variety*				
Sufficient (ref)	1.00		1.00	
Poor	1.29 [0.95–1.76]	0.102	1.23 [0.91–1.65]	0.180
*Exercise Habits (Days/Week)*				
Inactive (0–1) (ref)	1.00		1.00	
Mildly Active (2–3)	1.08 [0.81–1.45]	0.604	1.08 [0.79–1.48]	0.628
Moderately Active (4–5)	0.56 [0.28–1.13]	0.105	0.51 [0.23–1.12]	0.092
Very Active (6–7)	0.86 [0.52–1.43]	0.553	0.85 [0.50–1.45]	0.558
***Physical Violence***				
*Hours of Sleep/Night*				
1–4	0.62 [0.14–2.80]	0.534	0.67 [0.15–2.91]	0.589
5–6	0.93 [0.57–1.53]	0.788	0.99 [0.56–1.77]	0.986
7–8 (ref)	1.00		1.00	
9+	1.05 [0.69–1.60]	0.828	1.06 [0.70–1.63]	0.772
*Diet Variety*				
Sufficient (ref)	1.00		1.00	
Poor	0.96 [0.69–1.34]	0.810	0.90 [0.65–1.24]	0.519
*Exercise Habits (Days/Week)*				
Inactive (0–1) (ref)	1.00		1.00	
Mildly Active (2–3)	1.14 [0.75–1.73]	0.550	1.41 [0.90–2.22]	0.135
Moderately Active (4–5)	0.66 [0.26–1.65]	0.373	0.74 [0.30–1.87]	0.527
Very Active (6–7)	1.03 [0.53–2.00]	0.925	1.26 [0.69–2.31]	0.456
***Sexual Violence***				
*Hours of Sleep/Night*				
1–4	0.37 [0.08–1.76]	0.213	0.37 [0.08–1.79]	0.218
5–6	0.92 [0.65–1.32]	0.669	0.93 [0.63–1.37]	0.715
7–8 (ref)	1.00		1.00	
9+	0.84 [0.52–1.35]	0.468	0.79 [0.47–1.35]	0.387
*Diet Variety*				
Sufficient (ref)	1.00		1.00	
Poor	**1.64 [1.25–2.16]**	<0.001*	**1.57 [1.21–2.05]**	0.001*
*Exercise Habits (Days/Week)*				
Inactive (0–1) (ref)	1.00		1.00	
Mildly Active (2–3)	0.96 [0.65–1.42]	0.830	0.99 [0.64–1.52]	0.952
Moderately Active (4–5)	1.09 [0.63–1.90]	0.759	1.00 [0.54–1.86]	0.999
Very Active (6–7)	1.03 [0.55–1.91]	0.935	1.13 [0.61–2.08]	0.702
***Physical or Sexual Violence***				
*Hours of Sleep/Night*				
1–4	0.40 [0.11–1.48]	0.168	0.40 [0.11–1.49]	0.172
5–6	0.96 [0.65–1.41]	0.836	0.98 [0.65–1.48]	0.912
7–8 (ref)	1.00		1.00	
9+	0.91 [0.63–1.31]	0.603	0.88 [0.60–1.31]	0.540
*Diet Variety*				
Sufficient (ref)	1.00		1.00	
Poor	1.31 [1.00–1.72]	0.051	1.25 [0.96–1.62]	0.100
*Exercise Habits (Days/Week)*				
Inactive (0–1) (ref)	1.00		1.00	
Mildly Active (2–3)	0.99 [0.72–1.38]	0.971	1.10 [0.76–1.61]	0.608
Moderately Active (4–5)	0.94 [0.52–1.69]	0.834	0.92 [0.49–1.74]	0.806
Very Active (6–7)	1.05 [0.55–1.97]	0.889	1.18 [0.64–2.19]	0.596

Note: Adjusted models included age, migration status, education level, employment status, relationship status, alcohol consumption, and childhood experience of violence. Each regression used the full sample size (*n* = 754).

*boldface indicates statistical significance at α = 0.05.

^†^*p*-value calculated from Wald test.

^◊^the likelihood ratio test for an overall variable significance.

## Discussion

The overall prevalence of IPV perpetration in the sample was 79.4% for controlling behaviours, 30.6% for economic abuse, 47.3% for emotional abuse, 16.4% for physical abuse, 23.3% for sexual violence, and 32.1% for the combined physical and/or sexual violence category. The results were quite varied between different types of IPV, but some trends emerged. These trends should be considered in the narrative surrounding male-perpetration of IPV in the sub-Saharan context, as well as in the development of future IPV interventions. Mildly active lifestyles decreased the risk of IPV perpetration for economic abuse, but moderate and high levels of exercise had no effect. Inadequate sleep (1–4 h per night) demonstrated a protective effect for all types of IPV compared to sufficient sleep (7–8 h per night), and poor diet variety was generally associated with increased IPV perpetration risk for most types (aOR ≥1.20 for all types except physical violence). This data suggests there may be a weak connection between some healthy lifestyle factors and certain types of IPV perpetration, although the evidence is not overwhelmingly supportive and should be considered alongside other theories and findings.

The prevalence of male perpetrated IPV in this study was relatively consistent with what had been previously recorded in women-focused studies taking place in the same area; however, prevalence of emotional abuse and controlling behaviours were both approximately 10% higher than what the women enrolled in the MAISHA Trial had reported [1,4,11]. Some literature evidence also already existed regarding diet and sleep, although nearly all of it came from female studies [13,20,28].

In the case of diet, all comparative studies focused only on food insecurity, which may have different implications in IPV versus variety alone [29]. This study focused on only one aspect of food insecurity as a proxy for poor diet, so comparisons to previous literature were made cautiously. Poor food variety was associated with a higher risk of IPV perpetration for every type except for physical IPV, but was significant only for sexual IPV. These results are not supported by findings from Hatcher et al., where food insecurity was strongly associated with combined physical and/or sexual IPV perpetration in South Africa [29]. Two studies also found strong positive connections for physical and emotional IPV with food insecurity among women, but these studies took place outside of the sub-Saharan Africa context [13,28]. The association between previously reported types of IPV and food insecurity is unclear, and may be bidirectional; however, food insecurity contributes to overall household stress which follows the stress to aggression pathway explanation [13,24,28]. Diet also plays a prominent role in overall health and is implicated in various metabolic pathways in the body, including those related to stress. Consuming a sufficiently varied diet (which is often a healthier diet) could therefore play a role in lowering overall stress and in turn, aggression [24,43]. This theoretical pathway and association might help explain the findings, but due to diet’s close association with food insecurity in this study, and the complex relationship of food insecurity, poverty, stress, and IPV, future investigations should focus on further separating dietary patterns and micro/macronutrient consumption from food insecurity before solid conclusions can be drawn.

Sleep produced varying and unexpected results with IPV perpetration. While amounts of sleep other than the recommended 7–8 h were expected to be associated with increased IPV perpetration, inadequate sleep (1–4 h per night) appeared protective for emotional IPV [19]. This contradicts both the hypothesis and previous findings in the literature, where strong associations between poor sleep and both perpetration and experience of IPV have been established [14,33,44,45]. One plausible explanation might be that men who slept so little spent less time with their partners or in the home due to work or other behaviours and, therefore, may have had less opportunity to perpetrate IPV. It is also important to note that previous studies focused on older demographics than this study, which may influence results as younger populations may have different baseline sleep patterns and needs. Sensitivity analysis demonstrated that changing the sleep variable to dichotomous – sufficient sleep (≥7 h per night) or insufficient sleep (<7 h per night) – eliminated any notable associations between sleep and IPV perpetration. The lack of positive associations between sleep and IPV perpetration are surprising, especially due to the close link with poor mental health and the previously identified mediating relationship in IPV [33]. Considered alongside these results, future studies may investigate the temporality of poor sleep and IPV perpetration patterns. Additionally, evaluating proportion of time spent in the home or with intimate partners, and amount of direct communication in relationships, in connection to IPV perpetration, may also be beneficial as this could be an underlying factor to the relationship arising from inadequate sleep.

While most elements of this study are under-investigated in the field of IPV, especially from the male perpetration perspective, including exercise in the analysis was novel and the results were notable. Economic abuse had significant associations with increased physical activity. The risk of economic IPV perpetration was reduced with regular exercise habits. It is possible that men participating in exercise and recreation had lower overall stress levels, and therefore, were at lower risk of manifesting that stress into violence against their partners, which would support the theoretical pathway drawn in the conceptual diagram (Figure 1). This association is not well supported by sexual, physical, and physical or sexual IPV results where exercise does not appear to have any association and is contradicted by the results of the controlling behaviour models. In controlling behaviour, exercise appears to increase risk of perpetration; however, this could be due to the high prevalence of controlling behaviours (~80%) in the sample compared to other types of IPV. Another possible explanation for the protective effects of mild exercise may come from community-level factors rather than individual, but this connection was not seen in the more active groups. Weak community links are known to increase risk of IPV perpetration, and sports and recreation may strengthen these connections [46]. Men who participate in regular exercise may also feel more confident in their masculinity and feel less pressured to establish a dominant role in their relationships through economic or emotional abuse [15,47]. Physical activity may play a role in reducing IPV perpetration and maintaining healthy communities and individuals. This novel finding would benefit from further investigation in other Tanzanian and LMIC populations before being considered for intervention programmes.

The analysis provided evidence both in support of and in contrast to the original hypothesis, that a healthy lifestyle decreases the risk of IPV perpetration. This result is not particularly surprising due to the multidimensional conceptualisation between diet, sleep, and exercise, and IPV perpetration. Other variables and factors not related to a physically healthy lifestyle may better describe male perpetration behaviours, e.g. patriarchal structures, resource theory (related to economic stress and availability), or feminist theory (related to women’s empowerment) [48]. The results from this study are specific to the cultural and geographical context of Mwanza, Tanzania. They can reasonably be extended to populations represented in the sample, such as the rest of Tanzania and even sub-Saharan Africa if limitations are considered. Because of the unique and intricate relationship IPV has with communities, extending the results to other LMICs should be done with caution.

### Strengths and limitations

The questionnaire was derived from validated surveys and underwent rigorous testing and approval to ensure high levels of reliability. Additionally, there were no missing data in any of the variables included, resulting in a high level of data quality. Given the nature of IPV, disclosure rates may be lower than reality, which could bias the results as IPV perpetration prevalence may be underestimated. The use of electronic responses for IPV outcomes attempted to combat some of this bias, but false responses were still possible and expected. Including only the past 12 months in perpetration questions was intended to keep recall bias to a minimum. As this was a secondary analysis of the data and the research question was defined following data collection, unfortunately, no questions in the survey directly related to diet and a proxy variable regarding variety of available foods had to be used. Food insecurity has connections to IPV through pathways other than poor diet that may have influenced the observed relationships [30,31]. Due to the wording of the diet question to include household members, there may also be uncertainty as to whether the respondents themselves were affected and may therefore be overestimating the effect of diet.

Exercise was measured as number of days per week with at least 10 min of vigorous physical activity, meaning there was no way to determine total exercise time, which may be a more accurate measure of sufficient physical activity [34]. Additional limitations include a lack of established temporality (due to the cross-sectional nature) and the differences in relationship types. While we did control for relationship type in the adjusted model (Model 2), habitation patterns and numbers of partners could also impact IPV perpetration patterns which were not accounted for.

## Conclusion

The primary goal of this work was to contribute to the holistic understanding of male perpetrated IPV within the sub-Saharan African context. The intention was to identify potential risk factors and provide evidence for development of future male-targeted IPV intervention strategies, and context for site-specific tailored programmes in Tanzania and sub-Saharan Africa [9,18]. The findings from this study are indicative that physical health approaches may play a weak role in IPV perpetration but may not be as important as mental health and relationship dynamics. Some notable connections between healthy lifestyle behaviours and IPV perpetration were presented in this work, including the potential role of regular exercise in reducing economic abuse perpetration. Including these results in the field of literature and considering them in future interventions is important to make informed decisions and develop effective intervention strategies that reduce IPV levels, help reach SDGs, and provide better outcomes for all involved parties.

## Data Availability

The dataset generated and analysed for this study is not publicly available due to privacy and data protection measures.
